# Hepatitis B Virus Pre-S Gene Deletions and Pre-S Deleted Proteins: Clinical and Molecular Implications in Hepatocellular Carcinoma

**DOI:** 10.3390/v13050862

**Published:** 2021-05-08

**Authors:** Yueh-Te Lin, Long-Bin Jeng, Wen-Ling Chan, Ih-Jen Su, Chiao-Fang Teng

**Affiliations:** 1 Cancer Genome Research Center, Chang Gung Memorial Hospital, Taoyuan 333, Taiwan; dawnbread1207@gmail.com; 2 Organ Transplantation Center, China Medical University Hospital, Taichung 404, Taiwan; longbin.cmuh@gmail.com; 3 Department of Bioinformatics and Medical Engineering, Asia University, Taichung 413, Taiwan; wlchan@asia.edu.tw; 4 Epigenome Research Center, China Medical University Hospital, Taichung 404, Taiwan; 5 Department of Biotechnology, Southern Taiwan University of Science and Technology, Tainan 710, Taiwan; suihjen0704@stust.edu.tw; 6 Graduate Institute of Biomedical Sciences, China Medical University, Taichung 404, Taiwan; 7 Research Center for Cancer Biology, China Medical University, Taichung 404, Taiwan

**Keywords:** hepatocellular carcinoma, hepatitis B virus, pre-S gene deletions, pre-S deleted proteins, biomarkers, targets

## Abstract

Hepatocellular carcinoma (HCC) is one of the most frequent and fatal human cancers worldwide and its development and prognosis are intimately associated with chronic infection with hepatitis B virus (HBV). The identification of genetic mutations and molecular mechanisms that mediate HBV-induced tumorigenesis therefore holds promise for the development of potential biomarkers and targets for HCC prevention and therapy. The presence of HBV pre-S gene deletions in the blood and the expression of pre-S deleted proteins in the liver tissues of patients with chronic hepatitis B and HBV-related HCC have emerged as valuable biomarkers for higher incidence rates of HCC development and a higher risk of HCC recurrence after curative surgical resection, respectively. Moreover, pre-S deleted proteins are regarded as important oncoproteins that activate multiple signaling pathways to induce DNA damage and promote growth and proliferation in hepatocytes, leading to HCC development. The signaling molecules dysregulated by pre-S deleted proteins have also been validated as potential targets for the prevention of HCC development. In this review, we summarize the clinical and molecular implications of HBV pre-S gene deletions and pre-S deleted proteins in HCC development and recurrence and highlight their potential applications in HCC prevention and therapy.

## 1. Introduction

Hepatocellular carcinoma (HCC) is the predominant histological type of primary liver cancer, accounting for up to 90% of all cases [1,2]. Despite considerable progress made in the detection, prevention, and therapy of HCC, it remains the sixth most prevalent and the third most lethal human cancer worldwide, causing approximately 800,000 deaths every year [3,4]. Moreover, although curative surgical resection is available for some patients, the recurrence rate of HCC is up to 70% within five years after surgery, resulting in poor patient survival [5,6]. Therefore, understanding the pathological mechanisms of HCC development and recurrence is important for developing promising diagnostic preventive and therapeutic interventions to improve patient outcomes.

Among the risk factors for HCC development, chronic hepatitis B virus (HBV) infection plays a major role, contributing to over 50% of all cases worldwide [7,8]. Several indirect and direct mechanisms have been proposed to explain HBV-mediated HCC development, including the chronic hepatitis and regenerative hyperplasia caused by the host immune system attacking HBV-infected hepatocytes, the genomic instability and insertional mutagenesis induced by the integration of the HBV DNA genome into the host cell genome, and the oncogenic activities exhibited by HBV gene products such as the HBV X protein and pre-S deleted proteins [9,10]. Therefore, targeting the mechanisms underlying HBV-induced tumorigenesis is a promising strategy to discover potential biomarkers and targets for HCC prevention and therapy.

**Figure 1:**
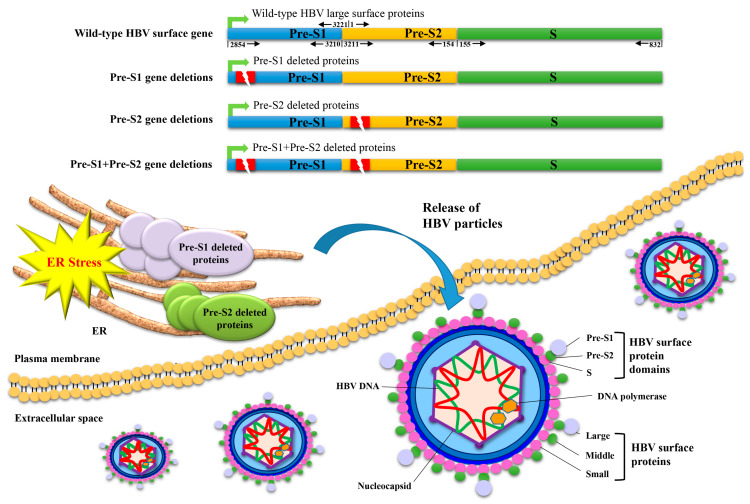
Schematic representation of HBV pre-S gene deletions and the expression and ER retention of pre-S deleted proteins. The HBV surface gene consists of three gene segments (pre-S1, pre-S2, and S) and expresses three different sizes of surface proteins (small, middle, and large) that are, respectively, composed of domains encoded from the S segment, the pre-S2 and S segments, and all three gene segments, collectively constituting the envelope proteins of viral particles. The numbers shown above and below the wild-type surface gene indicate the nt positions of each intact gene segment in the circular HBV DNA genome, which starts at nt 1 and ends at nt 3221 in a clockwise direction. Deletion mutations (indicated as red rectangle boxes with a lightning symbol) that occur solely in the pre-S1 or pre-S2 gene segment lead to the expression of pre-S1 or pre-S2 deletion mutants of large surface proteins, respectively. Unlike wild-type large surface proteins that are released as constituents of the envelope proteins of HBV particles from host cells, both pre-S1 and pre-S2 deleted proteins are mainly retained in the host cell organelle ER and induce ER stress. In addition, deletion mutations that occur concurrently in the pre-S1 and pre-S2 gene segments (the so-called pre-S1 + pre-S2 gene deletions) may lead to the expression of large surface proteins harboring both pre-S1 and pre-S2 deletions (the so-called pre-S1 + pre-S2 deleted proteins), which may act in a manner like pre-S1 and pre-S2 deleted proteins—although the exact molecular function remains to be clarified. The HBV DNA polymerase associated with the viral DNA genome and the nucleocapsid encompassing the viral DNA genome are also shown in the diagram. Abbreviations: HBV, hepatitis B virus; ER, endoplasmic reticulum.

The HBV surface gene contains three gene segments (pre-S1, pre-S2, and S) and expresses three different sizes of surface proteins (small, middle, and large), respectively, composed of domains encoded from the S segment, the pre-S2 and S segments, and all three gene segments, which collectively constitute the envelope proteins in viral particles (Figure 1) [11,12]. Several naturally occurring deletion mutations over the pre-S1 and/or pre-S2 gene segments (the so-called pre-S gene deletions) have been clinically identified and lead to the expression of large surface proteins that harbor deletions in the pre-S1 and/or pre-S2 domains (the so-called pre-S deleted proteins) (Figure 1) [13,14]. The presence of pre-S gene deletions in the blood and the expression of pre-S deleted proteins in the liver tissues of patients with chronic hepatitis B and HBV-related HCC have been significantly correlated with a higher risk of HCC development and recurrence after curative surgical resection, respectively [15,16]. Moreover, pre-S deleted proteins have been well demonstrated to promote HCC development through the activation of multiple oncogenic signaling pathways in hepatocytes, and their dysregulated signaling molecules have been shown to have potential as preventive targets for HCC [17,18].

In this review, we summarize the evidence supporting the clinical significance and molecular mechanisms of HBV pre-S gene deletions and pre-S deleted proteins in HCC development and their recurrence as well as underscore their potential implications in HCC prevention and therapy.

## 2. The Presence of HBV Pre-S Gene Deletions in the Blood of Patients with Chronic Hepatitis B Is Associated with Liver Disease Progression and Higher Incidence of Liver Cirrhosis and HCC Development

**Figure 2:**
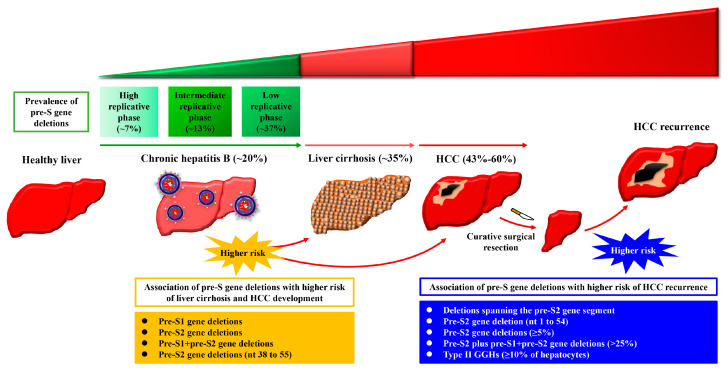
Schematic summary of the clinical association of HBV pre-S gene deletions with liver disease progression and HCC development and recurrence. In patients with chronic HBV infection, the prevalence of overall pre-S gene deletions (including pre-S1, pre-S2, and pre-S1 + pre-S2 gene deletions) in the blood gradually increases with liver disease progression from the high to intermediate to low replicative phases of chronic hepatitis B, to liver cirrhosis, and reaches the highest level at the stage of HCC development. The presence of pre-S gene deletions (pre-S1, pre-S2, and/or pre-S1 + pre-S2 gene deletions) or pre-S2 gene deletions between nts 38 and 55 (nt 38 to 55) predicts higher incidence of liver cirrhosis and HCC development. Moreover, in patients with HBV-related HCC, the presence of deletions spanning the pre-S2 gene segment, pre-S2 gene deletion at nts 1 to 54 (nt 1 to 54), pre-S2 gene deletions at ≥5%, or pre-S2 plus pre-S1+pre-S2 gene deletions at >25% in the blood or type II GGHs comprising ≥10% of hepatocytes in the liver tissues predicts the higher risk of HCC recurrence after curative surgical resection. Abbreviations: HCC, hepatocellular carcinoma; GGHs, ground glass hepatocytes.

Several retrospective studies have revealed that the prevalence of pre-S gene deletions in the blood of patients gradually increases during disease progression from chronic hepatitis B (from the high to intermediate to low replicative phases of HBV) to liver cirrhosis to HCC (Figure 2). Fan et al. and Shen et al. showed that the prevalence of overall pre-S gene deletions (including pre-S1, pre-S2, and pre-S1 + pre-S2 gene deletions) was about 7% at the high replicative phase, 13% at the intermediate replicative phase, and 37% at the low replicative phase of HBV in the blood of patients with chronic hepatitis B [19,20]. Choi et al., Li et al., and Zhao et al. further showed that the prevalence rates of overall pre-S gene deletions were about 20%, 35%, and 43% in the blood of patients with chronic hepatitis B, liver cirrhosis, and HCC, respectively [21,22,23]. Jia et al. also showed that the prevalence of overall pre-S gene deletions was significantly higher in HCC patients than in chronic hepatitis B patients [24]. In another cohort study, Shen et al. showed that the prevalence of overall pre-S gene deletions in the blood of HCC patients was even up to 60% [20]. Furthermore, Li et al. and Zhao et al. showed that the pre-S gene deletions of small size (≤99 nucleotides (nts)) or the pre-S2 gene deletions were more frequently detected in patients with HCC than in patients without [22,23].

In addition, Chen et al. and Sinn et al. conducted prospective studies, showing that the five-year cumulative incidence of liver cirrhosis and HCC development was about fivefold higher in chronic hepatitis B patients with pre-S gene deletions (pre-S1, pre-S2, and/or pre-S1 + pre-S2 gene deletions) in the blood than in patients without [25,26]. The presence of pre-S gene deletions in the blood was evaluated as an independent risk factor for liver cirrhosis and HCC development [25,26]. Moreover, Cohen et al. showed that the presence of pre-S2 gene deletions between nts 38 and 55 (a 12 nt, 15-nt, or 18 nt deletion) in the blood independently predicted HCC development [27]. Collectively, these results suggest a clinical correlation between pre-S gene deletions and HBV-related liver cirrhosis and HCC development.

## 3. The Presence of HBV Pre-S Gene Deletions in the Blood of Patients with HCC Is Associated with Higher Risk of HCC Recurrence after Curative Surgical Resection

The clinical correlation between pre-S gene deletions and HCC recurrence has been validated in several retrospective studies (Figure 2). Li-Shuai et al. showed that HCC patients with pre-S gene deletions (especially pre-S2 gene deletions) in the blood had a significantly higher rate of HCC recurrence after curative surgical resection than patients without [28]. Yen et al. further showed that higher percentages of pre-S2 gene deletions (≥5%) in the blood of HCC patients were correlated with lower rates of overall and recurrence-free survival after curative surgical resection [29]. The presence or percentage of pre-S2 gene deletions in the blood was determined as an independent prognostic factor for HCC recurrence [28,29]. Furthermore, Teng et al. showed that either the presence of deletions spanning the pre-S2 gene segment or a certain percentage of pre-S2 plus pre-S1 + pre-S2 gene deletions (>25%), or a combination of both factors in the blood of HCC patients, was independently associated with higher risk of HCC recurrence after curative surgical resection [30,31,32]. Additionally, Teng et al. showed that the pre-S2 gene deletion at nts 1 to 54 (a 54 nt deletion) was the most predominant region of pre-S gene deletions in the blood of HCC patients and independently predicted HCC recurrence after curative surgical resection [33]. Considering that this pre-S2 gene deletion region (nt 1 to 54) coincides with the B-and T-cell epitopes of HBV large surface proteins [34,35], pre-S2 deleted proteins may emerge as immune escape mutants, possibly conferring their growth advantage and explaining their strong association with HCC recurrence.

## 4. The Expression of Pre-S Deleted Proteins in the Liver Tissues of Patients with HCC Is Associated with Higher Risk of HCC Recurrence after Curative Surgical Resection

**Figure 3:**
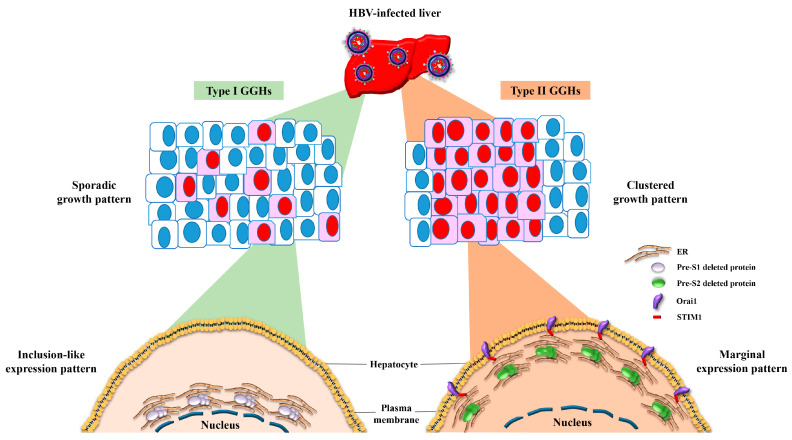
Schematic representation of the growth patterns of GGHs and the expression patterns of HBV pre-S deleted proteins. In the liver tissues of patients with chronic HBV infection, two distinct types of GGHs (type I and type II) are identified as HBV-infected hepatocytes that express pre-S1 and pre-S2 deleted proteins, respectively. Type I GGHs display a scattered or sporadic growth pattern and express pre-S1 deleted proteins in a globular or inclusion-like pattern due to the accumulation of pre-S1 deleted proteins in the ER, whereas type II GGHs display a clustered growth pattern and express pre-S2 deleted proteins in a marginal pattern due to the accumulation of pre-S2 deleted proteins in the ER followed by STIM1-and Orai1-mediated connection of the ER and plasma membrane. Abbreviations: HBV, hepatitis B virus; GGHs, ground glass hepatocytes; ER, endoplasmic reticulum; STIM1, stromal interaction molecule 1; Orai1, calcium release-activated calcium modulator 1.

Ground glass hepatocytes (GGHs) have been identified as HBV-infected hepatocytes that harbor pre-S gene deletions and express pre-S deleted proteins in the liver tissues of patients with chronic HBV infection (Figure 3) [13,14]. Depending on the type of pre-S deleted proteins expressed, GGHs are classified into two distinct types (type I and type II). Type I GGHs grow sporadically and express a globular or inclusion-like pattern of large surface proteins that harbor deletions in the pre-S1 domain (the so-called pre-S1 deleted proteins) whereas type II GGHs grow in clusters and express a marginal pattern of large surface proteins that harbor deletions in the pre-S2 domain (the so-called pre-S2 deleted proteins) (Figure 3) [13,14]. The retrospective studies conducted by Tsai et al. showed that higher expression scores of type II GGHs (≥10% of hepatocytes) in the liver tissues of HCC patients were independently associated with shorter overall and recurrence-free survival after curative surgical resection, no matter whether patients received pre-surgical anti-HBV treatment, supporting the clinical correlation between pre-S deleted proteins and HCC recurrence [36,37].

## 5. Both HBV Pre-S1 and Pre-S2 Deleted Proteins Activate Endoplasmic Reticulum (ER) Stress-Dependent Signaling Pathways to Induce DNA Damage and to Promote Growth and Proliferation in Hepatocytes In Vitro and In Vivo

**Figure 4:**
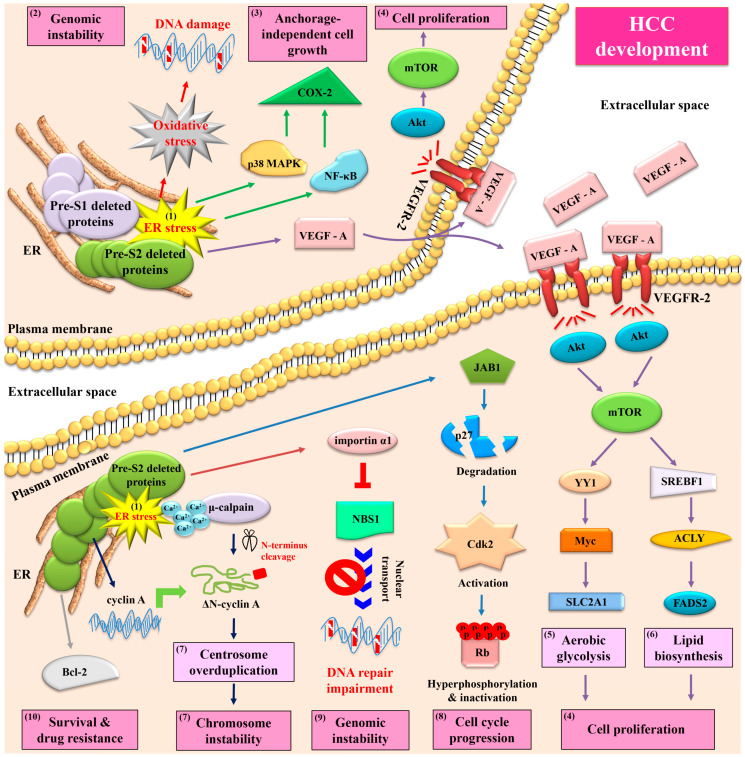
Schematic summary of signaling pathways activated by HBV pre-S deleted proteins and their implications in HCC development. In chronic HBV infection, both pre-S1 and pre-S2 deleted proteins accumulate in the ER and induce ER stress in hepatocytes (as highlighted by (1)). Through the mediation of ER stress, both types of pre-S deleted proteins can initiate three oncogenic signaling pathways, one involving oxidative stress to induce DNA damage and genomic instability (as highlighted by (2)), another involving NF-κB/p38 MAPK/COX-2 to enhance anchorage-independent cell growth (as highlighted by (3)), and the other involving VEGF-A/VEGFR-2/Akt/mTOR to promote cell proliferation (as highlighted by (4)). Through the mediation of mTOR activation, pre-S deleted proteins can further initiate two metabolism-related signaling pathways; one involving YY1/Myc/SLC2A1 to stimulate aerobic glycolysis (as highlighted by (5)) and the other involving SREBF1/ACLY/FADS2 to increase lipid biosynthesis (as highlighted by (6)), collectively contributing to cell proliferation. Furthermore, pre-S2 deleted proteins can additionally initiate four ER stress-dependent or -independent signaling pathways: one involving Ca^2+^/μ-calpain/ΔN-cyclin A to induce centrosome overduplication and chromosome instability (as highlighted by (7)), one involving JAB1/p27/Cdk2/Rb to promote cell cycle progression (as highlighted by (8)), one involving importin α1/NBS1 to inhibit DNA repair (as highlighted by (9)), and the other involving Bcl-2 to enhance cell survival and drug resistance (as highlighted by (10)). The combined effects of pre-S deleted proteins on DNA damage and genomic instability, anchorage-independent growth and proliferation, aerobic glycolysis and lipid biosynthesis, centrosome overduplication and chromosome instability, cell cycle progression, and cell survival result in HCC development. Abbreviations: HBV, hepatitis B virus; ER, endoplasmic reticulum; NF-κB, nuclear factor-κB; p38 MAPK, p38 mitogen-activated protein kinase; COX-2, cyclooxygenase-2; VEGF-A, vascular endothelial growth factor-A; VEGFR-2, vascular endothelial growth factor receptor-2; Akt, protein kinase B; mTOR, mammalian target of rapamycin; YY1, Yin Yang 1; SLC2A1, solute carrier family 2 (facilitated glucose transporter) member 1; SREBF1, sterol regulatory element-binding transcription factor 1; ACLY, adenosine triphosphate citrate lyase; FADS2, fatty acid desaturase 2; Ca^2+^, calcium; ΔN-cyclin A, N-terminus truncated cyclin A; JAB1, Jun activation domain-binding protein 1; Cdk2, cyclin-dependent kinase 2; Rb, retinoblastoma protein; NBS1, Nijmegen breakage syndrome 1; Bcl-2, B cell lymphoma-2; HCC, hepatocellular carcinoma.

In contrast to the wild-type HBV large surface proteins that constitute the envelope proteins in viral particle assembly and mediate viral particle release from host cells, both pre-S1 and pre-S2 deleted proteins have been shown to preferentially accumulate in the host cell organelle ER, leading to an induction of ER stress (Figure 1) [13]. Through the mediation of ER stress, both types of pre-S deleted proteins have been demonstrated to activate multiple oncogenic signaling pathways in hepatocytes, eventually contributing to HCC development (Figure 4). Hsieh et al. showed that pre-S deleted proteins could cause oxidative-stress-induced (via reactive oxygen species) DNA damage in human hepatoma cell lines, in transgenic mouse livers, and in GGHs in HCC patients, potentially contributing to genomic instability [38]. Hung et al. showed that pre-S deleted proteins could induce the expression of cyclooxygenase-2 (COX-2) through the activation of nuclear factor-κB (NF-κB) and p38 mitogen-activated protein kinase (p38 MAPK) in human hepatoma cell lines, in transgenic mouse livers, and in GGHs in HCC patients, which enhanced anchorage-independent cell growth [39]. Yang et al. showed that pre-S deleted proteins could upregulate the expression and secretion of vascular endothelial growth factor (VEGF)-A, which could, in turn, bind to VEGF receptor-2 (VEGFR-2) in an autocrine or paracrine manner to activate the protein kinase B (Akt)/mammalian target of the rapamycin (mTOR) signaling pathway in human hepatoma cell lines, in transgenic mouse livers, and in GGHs in HCC patients, which promoted cell proliferation [40]. Furthermore, Teng et al. showed that through the mediation of mTOR activation, pre-S deleted proteins could further initiate two metabolism-related signaling pathways in human hepatoma cell lines and in transgenic mouse livers, one involving Yin Yang 1(YY1)/Myc/solute carrier family 2 (facilitated glucose transporter) member 1 (SLC2A1) to stimulate aerobic glycolysis and the other involving sterol regulatory element-binding transcription factor 1 (SREBF1)/adenosine triphosphate citrate lyase (ACLY)/fatty acid desaturase 2 (FADS2) to increase lipid biosynthesis, collectively contributing to cell proliferation [41,42].

## 6. HBV Pre-S2 Deleted Proteins Additionally Activate ER Stress-Dependent or Independent Signaling Pathways to Induce Centrosome Overduplication, Promote Cell Cycle Progression, Inhibit DNA Repair, and Enhance Survival and Drug Resistance in Hepatocytes In Vitro and In Vivo

Aside from the ER stress-dependent signaling pathways initiated by both types of pre-S deleted proteins, pre-S2 deleted proteins have been shown to activate additional signaling pathways with or without the mediation of ER stress (Figure 4). Wang et al. showed that pre-S2 deleted proteins could not only upregulate the expression of cyclin A at the transcription level in an ER stress-independent manner, but also induce calcium (Ca^2+^)-dependent protease μ-calpain-mediated cleavage of cyclin A through the mediation of ER stress, collectively resulting in the elevated production of N-terminus truncated cyclin A (ΔN-cyclin A) in human hepatoma cell lines, in transgenic mouse livers, and in GGHs in HCC patients [43,44]. Unlike its full-length counterpart, which was mainly detected in the cell nucleus, ΔN-cyclin A was predominantly located in the cytoplasm and induced centrosome overduplication, potentially contributing to chromosome instability [44]. Furthermore, Wang et al. showed that the induction of Ca^2+^ efflux from the ER lumen by pre-S2 deleted proteins could trigger the oligomerization of stromal interaction molecule 1 (STIM1) on the outer membrane of ER and the recruitment of the STIM1-resident ER toward the plasma membrane-localized Ca^2+^ release-activated Ca^2+^ modulator 1 (Orai1), resulting in Ca^2+^ influx into the cytoplasm and a consequent increase in the cytoplasmic Ca^2+^ concentration, further contributing to μ-calpain activation and its mediated centrosome overduplication [45]. The STIM1-and Orai1-mediated ER and plasma membrane connections may explain the marginal expression pattern of pre-S2 deleted proteins in the type II GGHs (Figure 3). In addition, Hsieh et al. and Hsu et al. showed that without the mediation of ER stress, pre-S2 deleted proteins could induce the degradation of cyclin-dependent kinase (Cdk) inhibitor p27 through zinc-dependent interaction with Jun activation domain-binding protein 1 (JAB1), leading to Cdk2-mediated hyperphosphorylation and the inactivation of the tumor suppressor retinoblastoma protein (Rb) in human hepatoma cell lines and in transgenic mouse livers, which promoted cell cycle progression [46,47]. Hsieh et al. further showed that pre-S2 deleted proteins could inhibit DNA double-strand break repair through interaction with importin α1 to block the nuclear transport of an essential DNA repair and recombination factor, Nijmegen breakage syndrome 1 (NBS1), in human hepatoma cell lines, in transgenic mouse livers, and in GGHs in HCC patients, leading to genomic instability [48]. Moreover, Hung et al. showed that pre-S2 deleted proteins could increase the expression of apoptosis regulator B cell lymphoma-2 (Bcl-2) at both the mRNA and protein levels in human hepatoma cell lines and transgenic mouse livers, resulting in enhanced survival and resistance to the chemotherapeutic drug 5-fluorouracil [49].

**Figure 5:**
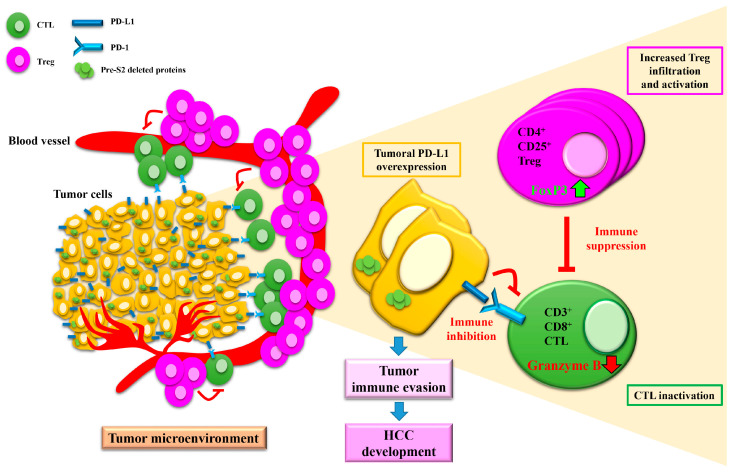
Schematic representation of the hypothetical model of HBV pre-S2 deleted proteins in the regulation of the tumor immune microenvironment in HCC. In the tumor microenvironment of HCC patients positive for pre-S2 deleted proteins, the expression of PD-L1 in tumor cells is elevated, the infiltration and activity of CD4^+^CD25^+^ Tregs are increased (as shown by increased cell number and forkhead box P3 (Foxp3) expression), and the activity of CD3^+^CD8^+^ CTLs is decreased (as shown by decreased granzyme B expression and secretion). Tregs have been shown to exert “pro-tumor” activity by suppressing the activity of “anti-tumor” CTLs [57]. In addition, PD-L1 expressed in tumor cells has been shown to interact with its receptor, PD-1, expressed in CTLs, to dampen the attack of tumor cells by CTLs [58]. The combined effects of tumoral PD-L1 overexpression, increased Treg infiltration and activation, and CTL inactivation favor tumor cell evasion from host immune surveillance, contributing to HCC development in patients with pre-S2 deleted proteins. Abbreviations: Treg, regulatory T cell; Foxp3, forkhead box P3; CTL, cytotoxic T cell; PD-L1, programmed death ligand 1; PD-1, programmed death 1; HCC, hepatocellular carcinoma.

Most recently, Teng et al. showed that HCC patients with pre-S2 gene deletions in the blood displayed higher numbers and activity levels of “pro-tumor” regulatory T cells (Tregs), similar numbers but lower activity levels of the “anti-tumor” cytotoxic T cells (CTLs), and higher expression levels of immune checkpoint molecule programmed death ligand 1 (PD-L1) but similar expression levels of its receptor programmed death 1 (PD-1) in tumor tissues than patients without did, suggesting a possible role of pre-S2 deleted proteins in the regulation of the tumor immune microenvironment in HBV-related HCC, although the exact mechanisms remain to be clarified (Figure 5) [50,51]. Considering that increased Tregs and decreased CTLs in number and activity in tumor tissues have been shown to promote tumor immune evasion and are associated with poor outcomes in HCC patients [52], suppressing Tregs or enhancing CTLs in number or activity in the tumor microenvironment may be a promising strategy for treating HCC patients who are positive for pre-S2 deleted proteins. Moreover, it has been shown that high PD-L1 expression in tumor tissues is correlated with a poor prognosis in HCC patients [53,54,55] and predicts a better response to PD-L1-based therapy in cancer patients [56]. Immune checkpoint inhibitors targeting PD-L1 may also hold promise as potential therapeutics for HCC patients with pre-S2 gene deletions.

## 7. HBV Pre-S2 Deleted Proteins Induce Malignant Transformation of Hepatocytes and Trigger HCC Development in a Transgenic Mouse Model

The ability of pre-S2 deletion mutant proteins to drive hepatocyte transformation and liver disease progression to HCC development has been well demonstrated. Wang et al. showed that pre-S2 deleted proteins could enhance proliferation, multinucleation, and anchorage-independent growth in untransformed human hepatocyte cell lines [43]. Furthermore, Teng et al. showed that transgenic mice that specifically expressed pre-S2 deleted proteins in the liver displayed several groups of liver pathologies (frequencies in two independent transgenic mouse lines after 30 months of follow-up): Group 1, chronic inflammation solely (11% to 24%); Group 2, hepatic steatosis solely (6% to 7%); Group 3, fibrosis solely (6% to 15%); Group 4, chronic inflammation and fibrosis (30% to 39%); Group 5, chronic inflammation, fibrosis, and hepatomegaly (3% to 12%); and Group 6, chronic inflammation, fibrosis, hepatomegaly, and HCC (12% to 15%) [59]. Consistent with the in vitro effect of pre-S2 deleted proteins in human hepatoma cell lines, ER stress and metabolic disturbance were also observed in the livers of the transgenic mice at an early stage before the development of liver pathologies, followed by a series of the activation of oncogenic signaling pathways during disease progression to HCC development [39,40,59,60].

## 8. Inhibition of Signaling Pathways Activated by HBV Pre-S Deleted Proteins Exhibits a Preventive Effect on Liver Pathology and HCC Development in a Transgenic Mouse Model

The efficacy of targeting signaling pathways activated by pre-S deleted proteins in ameliorating liver pathology and preventing HCC development has been validated in a transgenic mouse model by several studies. Teng et al. showed that a combination treatment of resveratrol and silymarin—two natural products extracted from grapes and milk thistles, respectively—could inhibit the formation of HCC in transgenic mouse livers through the suppressing activation of the mTOR/YY1/Myc/SLC2A1 signaling pathway [41]. Teng et al. also showed that treatment with a phytosome formulation of curcumin, a natural product extracted from turmeric, could improve chronic inflammation, decrease hepatic steatosis, and suppress HCC formation in transgenic mouse livers by inhibiting NF-κB and mTOR activation [61]. Furthermore, Hsieh et al. showed that treatment with suberoylanilide hydroxamic acid, a histone deacetylase inhibitor, could reduce cell proliferation, the nuclear/cytoplasmic ratio, and multinucleation in transgenic mouse livers through elevating the expression of thioredoxin-binding protein 2, which competes with pre-S2 deleted proteins for interaction with JAB1, thereby blocking p27 degradation [62].

## 9. Discussion

Although this review provides evidence supporting the clinical and molecular implications of HBV pre-S deleted proteins in HCC, some issues are still worth considering. First, type I and type II GGHs have been identified to harbor pre-S1 and pre-S2 deleted proteins, respectively [13,14]; however, these two types of GGHs also express wild-type HBV large surface proteins. Huang et al. showed that the expression of wild-type large surface proteins resulted in the formation of GGHs and sustained hepatocyte proliferation in the liver of transgenic mice [63]. Considering that the prevalence of pre-S gene deletions gradually increases during the progression of chronic HBV infection-related liver diseases and is associated with the higher incidence of liver cirrhosis and HCC development [21,22,23,24,25], it is reasonable to speculate that both type I and type II GGHs may evolve from the GGHs, which solely express wild-type large surface proteins; the expression of wild-type large surface proteins may initiate the formation of GGHs and sustain their proliferation, but the emergence of pre-S deleted proteins may confer a significant growth advantage to GGHs and promote their malignant transformation. Second, pre-S deleted proteins have been shown to activate multiple oncogenic signaling pathways [18], all of which are not induced by wild-type large surface proteins or are only induced to a much lesser extent. For example, Xu et al. showed that the large surface proteins retained within the secretory pathway, rather than the secretable form of large surface proteins, could cause ER stress in human hepatoma cell lines [64]. Wang et al. further showed that pre-S deleted proteins, but not wild-type large surface proteins, preferentially accumulated in the ER and induced ER stress [13]. However, Li et al. showed that expression of wild-type large surface proteins was sufficient to induce cytokinesis failure, leading to hepatocyte hyperploidy [65]. These findings therefore suggest that pre-S deleted proteins may exhibit a broader range and stronger activation of signaling pathways than wild-type large surface proteins in GGHs. Finally, but no less importantly, to date the majority of the cellular experiments performed to elucidate the signaling pathways activated by pre-S deleted proteins have been carried out in human hepatoma cell lines. Although most of the signaling pathways have also been validated in transgenic mouse livers and in GGHs in HCC patients, it remains to be verified whether pre-S deleted proteins exert the same effect in untransformed human hepatocyte cell lines.

## 10. Conclusions

This review highlights that the presence of HBV pre-S gene deletions in the blood and the expression of pre-S deleted proteins in the liver tissues of patients represent valuable biomarkers for HCC development and recurrence after curative surgical resection. Targeting the molecular mechanisms underlying tumorigenesis induced by pre-S deleted proteins is a promising strategy for preventing HBV-related chronic liver diseases and HCC.
